# C-Phycocyanin and *Lycium barbarum* Polysaccharides Protect against Aspirin-Induced Inflammation and Apoptosis in Gastric RGM-1 Cells

**DOI:** 10.3390/nu14235113

**Published:** 2022-12-01

**Authors:** Yu-Chen Liu, Chun-Chao Chang, Hirofumi Matsui, Jane C.-J. Chao

**Affiliations:** 1 School of Nutrition and Health Sciences, Taipei Medical University, 250 Wu-Hsing Street, Taipei 110301, Taiwan; 2 Division of Gastroenterology and Hepatology, Department of Internal Medicine, Taipei Medical University Hospital, 252 Wu-Hsing Street, Taipei 110301, Taiwan; 3 Division of Gastroenterology and Hepatology, Department of Internal Medicine, School of Medicine, Taipei Medical University, 250 Wu-Hsing Street, Taipei 110301, Taiwan; 4 TMU Research Center for Digestive Medicine, Taipei Medical University, 250 Wu-Hsing Street, Taipei 110301, Taiwan; 5 Division of Gastroenterology, Faculty of Medicine, University of Tsukuba, 1-1-1 Tennodai, Tsukuba 305-8575, Japan; 6 Master Program in Global Health and Health Security, Taipei Medical University, 250 Wu-Hsing Street, Taipei 110301, Taiwan; 7 Nutrition Research Center, Taipei Medical University Hospital, 252 Wu-Hsing Street, Taipei 110301, Taiwan

**Keywords:** apoptosis, aspirin, C-phycocyanin, extracellular signal-regulated kinase, inflammation, *Lycium barbarum* polysaccharides

## Abstract

Aspirin causes gastrotoxicity and damaged epithelial defense via cyclooxygenase inhibition. C-phycocyanin (CPC) and *Lycium barbarum* polysaccharides (LBP), an active ingredient of *Spirulina platensis* and wolfberry, respectively, exerted antioxidation, anti-inflammation, and/or immunoregulation. The actions of CPC and/or LBP on gastric damage induced by aspirin were explored in rat gastric mucosal RGM-1 cells. Gastric injury was performed by 21 mM aspirin for 3 h after the pretreatment of CPC and/or LBP (100–500 μg/mL) for 24 h in RGM-1 cells. Proinflammatory, anti-inflammatory, and apoptotic markers were examined by ELISA or gel electrophoresis and Western blotting. Cell viability and interleukin 10 (IL-10) were reduced by aspirin. Increased proinflammatory markers, caspase 3 activity, and Bax protein were observed in RGM-1 cells with aspirin treatment. Aspirin elevated nuclear factor-κB (NF-κB), extracellular signal-regulated kinase (ERK), p38, and c-Jun N-terminal kinase (JNK) activation, while CPC and/or LBP increased IL-10, and attenuated proinflammatory markers, Bax protein, NF-κB, and the activation of ERK and JNK. Therefore, CPC and/or LBP possess anti-inflammation by restraining the activation of the ERK signaling pathway, and LBP decreases apoptosis by suppressing the JNK signaling pathway activation in gastric RGM-1 cells with aspirin-induced epithelial damage.

## 1. Introduction

Aspirin, a non-steroidal anti-inflammatory drug (NSAID), has analgesic and anti-inflammatory characteristics. Low-dose aspirin (75–325 mg/day) has been used for cardiovascular protection and was demonstrated in clinical practice guidelines for established atherosclerotic cardiovascular disease and acute management of myocardial infarction [1]. However, long-term (>3 months) administration of low-dose aspirin was correlated with upper gastrointestinal side effects such as dyspepsia and peptic ulcer [2]. The acidity (pKa 3.5) and lipid solubility (log *P* = 1.15) of aspirin could impair the barrier of gastric mucosa [3]. In addition, aspirin inhibited the activity of cyclooxygenases (COX), thereby reducing the production of gastroprotective prostaglandins [3]. NSAIDs also generate free radicals and inhibit antioxidant enzymes in the process of inducing peptic ulcers [4]. The generation of free radicals could be caused by Bax, caspase-8, caspase-9, and caspase-3, which may further lead to apoptosis of gastrointestinal mucosal cells [5]. Previous studies have shown that under the induction of lipopolysaccharide, extracellular signal-regulated kinase (ERK) could be activated, and resulted in the inhibition of IκB kinase (IKK) phosphorylation and then the activation of nuclear factor-κB (NF-κB) pathway to promote the production of related inflammatory factors [6].

Phycobiliproteins, such as phycocyanin and phycocyanobilin, with similar structures to biliverdin and bilirubin [7], respectively, are biliprotein pigments of *Spirulina platensis* [8]. These proteins are divided into two categories: red phycoerythrin and blue phycocyanin, and the latter includes C-phycocyanin (CPC), R-phycocyanin, and allophycocyanin [8]. CPC is not toxic and carcinogenic and has been applied as a dye in food and cosmetics [8]. CPC also possesses antioxidant, anti-inflammatory, and free radical-eliminating characteristics [8]. The antioxidant capacity of phycocyanin was similar to biliverdin [7]. In addition, CPC has been found to stimulate cell migration into the wound site [9] and promote wound healing in animal experiments [10].

*Lycium barbarum L*. was also called wolfberry or goji berry and has been utilized for over 2300 years as one of the traditional Chinese herbal medicines [11]. There were 31 polysaccharides found in *Lycium barbarum*, which accounted for 5–8% of the dry weight [12]. The main monosaccharides were xylose, glucose, rhamnose, arabinose, fructose, trehalose, mannose, ribose, and galactose [12], and different compositions of *Lycium barbarum* polysaccharides (LBP) such as LBPA3, LBPB1, LBP-IV, and LpGp1 (-4) and possible structures with (1 → 3), (1 → 4), or (1 → 6) α- or β-glycosidic bond were reported previously [13]. LBP has been shown to own antioxidant and anti-cancer characteristics and protect the liver and small intestine from ischemia-reperfusion injury [14].

Aspirin has been applied to cardiovascular disease. However, chronic aspirin use could lead to gastric lesions. Although certain medications can be used to treat gastric damage caused by aspirin, they have several side effects. Our motivation was to use food ingredients to eliminate gastric damage caused by aspirin. CPC and LBP are both natural ingredients of edible food and have been proven to have antioxidant and anti-inflammatory effects. However, the actions of CPC and LBP on gastric damage caused by aspirin remained unexplored. Therefore, we studied whether CPC and/or LBP protected against gastric lesions in rat gastric mucosal RGM-1 cells damaged by aspirin.

## 2. Materials and Methods

### 2.1. Cells and Treatments

We bought rat normal gastric mucosa RGM-1 cells from RIKEN BioResource Center (Tsukuba, Ibaraki, Japan), and the morphology of RGM-1 cells was shown previously [15]. Cells were maintained in 20% fetal bovine serum, Dulbecco’s modified Eagle’s medium, and Ham’s F-12 mixture. The preparation of aspirin (Sigma Chemical Co., St. Louis, MO, USA) was referred to in the previous study [16]. Aspirin was dissolved in dimethyl sulfoxide and then added to the medium. CPC was provided by Far East Biotechnology. The spirulina powder was mixed with water for 24-h extraction followed by centrifugation, and the supernatant was collected and lyophilized. This lyophilized spirulina extract contained 24.4% phycocyanin (CPC and isophycocyanin), 35–45% polysaccharide, 10–20% non-phycocyanin protein, 5–8% moisture, and 10–12% ash. The treatment of LBP contained 40% LBP (GojiMax^®^ 40%, Priority Healthfood Corporation, New Taipei, Taiwan), which was analyzed by spectrophotometry. Gastric lesions were performed by aspirin (21 mM) for 3 h after the pretreatment of CPC and/or LBP (100–500 μg/mL) for 24 h in RGM-1 cells. Cells and the conditioned media were collected for further analyses. We used the colorimetric method to examine cell viability as the method of Saito et al. [17] using the MTS (3-(4,5-dimethylthiazol-2-yl)-5-(3-carboxymethoxyphenyl)-2-(4-sulfophenyl)-2H-tetrazolium, inner salt) kit (K300-2500, BioVision Inc., Milpitas, CA, USA). The viable cells were determined by colored formazan compound at 490–500 nm.

### 2.2. Proinflammatory and Anti-Inflammatory Markers and Regulators

The proinflammatory markers such as TNF-α (Cloud-Clone Corp., Houston, TX, USA), IL-6 (Cloud-Clone Corp.), and NF-κB (NF-κB (p65) Transcription Factor Assay Kit, Cayman Chemical Co., Ann Arbor, MI, USA) and anti-inflammatory marker such as IL-10 (Cloud-Clone Corp.) were examined by the ELISA kits. The absorbance of TNF-α, IL-6, and IL-10 in the conditioned medium was quantitated at 450 nm. The cells were washed with phosphate-buffered saline (PBS) with phosphatase inhibitors, added to the cell lysis buffer and NP-40 assay buffer, and centrifuged at 14,000× *g* for 30 s to precipitate the cell pellet. The cell pellet was dissolved in nuclear extraction buffer and centrifuged at 14,000× *g* for 10 min for NF-κB analysis. We finally measured the DNA binding activity of NF-κB in the nuclear fraction at 450 nm.

Protein expression of p-ERK, ERK, p-IκB-α, and IκB-α was determined by sodium dodecyl sulfate (SDS)-polyacrylamide gel electrophoresis (PAGE) and Western blotting. The cell suspension was applied to 10% SDS-PAGE at 40 mA for 1.5 h, and proteins were transferred to the nitrocellulose membrane by semi-dry electroblotting apparatus at 220 mA for 1 h and 20 min. The membrane was blocked in the blocking buffer (3% bovine serum albumin and Tris-buffered saline with Tween 20), incubated with primary antibody for p-ERK, ERK, p-IκB-α, or IκB-α (Santa Cruz Biotechnology Inc., Santa Cruz, CA, USA) and secondary antibody (Santa Cruz Biotechnology Inc.), and washed by Tris-buffered saline (TBS) and Tween 20 in between. The bands on the membrane were visualized using a commercial kit (Opti-ECL HRP Reagent Kit, Bioman Scientific Co., Ltd., New Taipei, Taiwan). Protein expression of β-actin was an internal control. The membrane was probed with different primary antibodies after the membrane was stripped by stripping buffer and washed by TBS and Tween 20 in between. The relative level was determined by chemiluminescence using an imaging system (UVP ChemiDoc-It 515 Imaging System Vision Works 8.18, UVP, LLC, Upland, CA, USA) and the software (Media Cybernetics, Inc., Rockville, MD, USA).

### 2.3. Apoptotic Markers and Regulators

Caspase 9 and caspase 3 activities were evaluated by the colorimetric kits (BioVision Inc., Milpitas, CA, USA). The cells were mixed with cell lysis buffer and centrifuged at 10,000× *g* for 1 min. Proteins were quantitated, added to the reaction buffer and chromophore DEVD-pNA (aspartyl-glutamyl-valyl-aspartyl-p-nitroanilide) at 37 °C for 2 h, and these activities were finally measured at 400 nm. The relative enzyme activity was expressed as % of the control group.

Apoptotic regulators such as Bax, Bcl-2, p-p38, p38, p-JNK, and JNK (BioVision Inc.) were isolated by 10% SDS-PAGE and determined by Western blotting mentioned above. The transferred proteins on the nitrocellulose membrane were identified by the corresponding primary antibodies (Santa Cruz Biotechnology Inc.) and incubated with the secondary antibodies and *chemiluminescence* reagent (Opti-ECL HRP Reagent Kit, Bioman Scientific Co., Ltd.). Protein expression of β-actin was an internal control. The membrane was stripped by the method described above for incubation with different primary antibodies. The image of protein bands was examined by the method described above.

### 2.4. Statistical Analysis

Data are expressed as mean ± standard deviation (SD). The statistical method was done by one-way analysis of variance (ANOVA), and the treatment effect was determined by Tukey’s multiple comparison test using GraphPad Prism 5.0 (GraphPad Software Inc., San Diego, CA, USA). The value of *p* < 0.05 was significant statistically.

## 3. Results

### 3.1. Cell Viability

**Figure 1:**
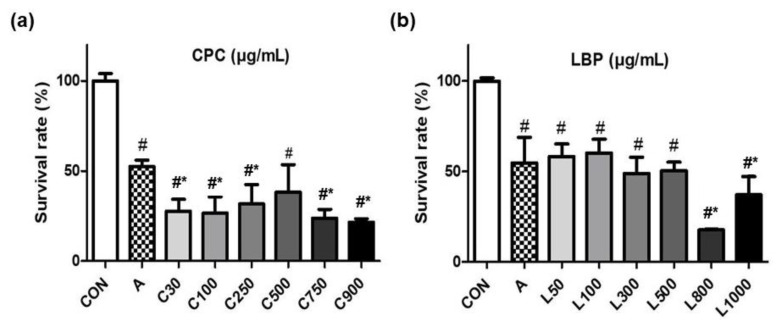
Effects of (**a**) C-phycocyanin (CPC) or (**b**) *Lycium barbarum* polysaccharides (LBP) on the cell survival rate of RGM-1 cells with aspirin-induced gastric damage. CON: control group, A: aspirin-induced group, C: CPC treated group, L: LBP treated group. Data are mean ± SD (*n* = 6). # *p* < 0.05 compared with the CON group. * *p* < 0.05 compared with the A group.

RGM-1 cells were pretreated with CPC (30–900 μg/mL) or LBP (50–1000 μg/mL) for 24 h and then induced lesions by 21 mM aspirin for 3 h. Cell viability was significantly reduced by pretreating with high doses of CPC (750 and 900 μg/mL) (Figure 1a) or LBP (800 and 1000 μg/mL) (Figure 1b). Considering not affecting cell viability by the pretreatment, 3 different doses (100, 250, and 500 μg/mL) for CPC or LBP were used in this study. Additionally, to explore whether the action of combined CPC and LBP was better, 250 μg/mL CPC and 250 μg/mL LBP (C + L) were used.

### 3.2. Effects of CPC and LBP on Proinflammatory and Anti-Inflammatory Markers

**Figure 2:**
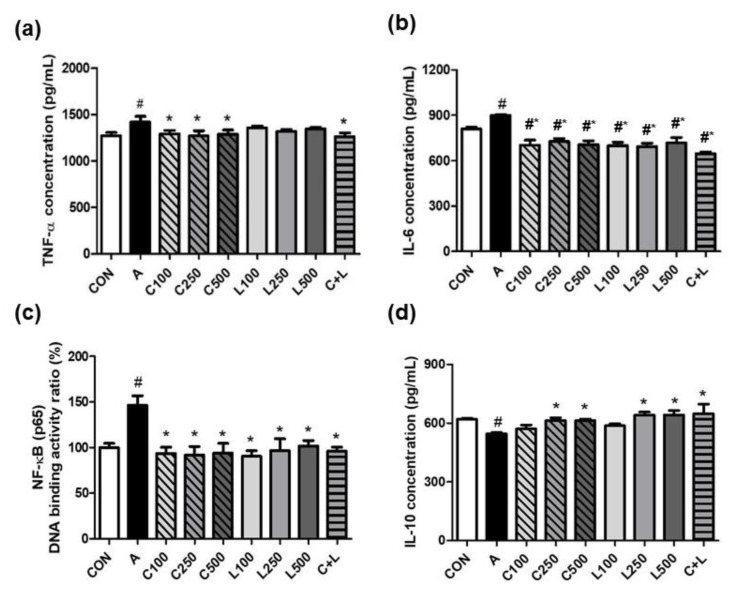
Effects of aspirin, CPC, and/or LBP on proinflammatory markers (**a**) TNF-α, (**b**) IL-6, (**c**) NF-κB (p65) DNA binding activity ratio, and anti-inflammatory marker (**d**) IL-10. CON: control group, A: aspirin-induced group, C: CPC treated group, L: LBP treated group. Data are mean ± SD (*n* = 3). # *p* < 0.05 compared with the CON group. * *p* < 0.05 compared with the A group.

Aspirin significantly increased the secretion of tumor necrosis factor-α (TNF-α) (1418 ± 64 pg/mL) (Figure 2a) and interleukin-6 (IL-6) (898 ± 7 pg/mL) (Figure 2b), and NF-κB (p65) DNA binding activity ratio (93.5% ± 6.9%) (Figure 2c) in RGM-1 cells. The treatment of CPC at 100, 250, or 500 μg/mL and C + L (250 μg/mL CPC and 250 μg/mL LBP) significantly decreased TNF-α concentration (1291 ± 39 pg/mL, 1269 ± 57 pg/mL, 1289 ± 43 pg/mL, and 1263 ± 41 pg/mL, respectively) compared with the aspirin-induced group. The treatment of CPC and/or LBP at all doses significantly attenuated IL-6 secretion and NF-κB (p65) DNA binding activity ratio compared with aspirin alone. Aspirin significantly reduced IL-10 secretion compared with the control group (Figure 2d). The treatment of CPC or LBP at higher doses (250 and 500 μg/mL) and C + L significantly increased IL-10 secretion compared with the aspirin-induced group.

### 3.3. Effects of CPC and LBP on Inflammatory Regulators

**Figure 3:**
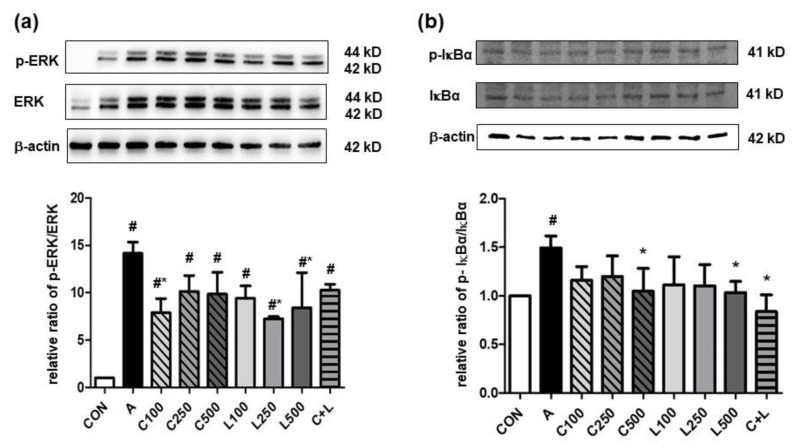
Effects of aspirin, CPC, and/or LBP on the relative ratio of (**a**) p-ERK/ERK (*n* = 3) and (**b**) p-IκBα/IκBα (*n* = 4). CON: control group, A: aspirin-induced group, C: CPC treated group, L: LBP treated group. Data are mean ± SD. # *p* < 0.05 compared with the CON group. * *p* < 0.05 compared with the A group.

Aspirin significantly elevated the relative ratio of the phosphorylated extracellular signal-regulated kinase (p-ERK)/ERK (Figure 3a) and phosphorylated IκB-α (p-IκB-α)/IκBα (Figure 3b). However, the treatment of 100 μg/mL CPC or 250 μg/mL and 500 μg/mL LBP decreased the relative ratio of p-ERK/ERK, and a high dose of CPC or LBP (500 μg/mL) and C + L treatments significantly decreased the relative ratio of p-IκBα/IκBα.

### 3.4. Actions of CPC and LBP on Apoptotic Markers

**Figure 4:**
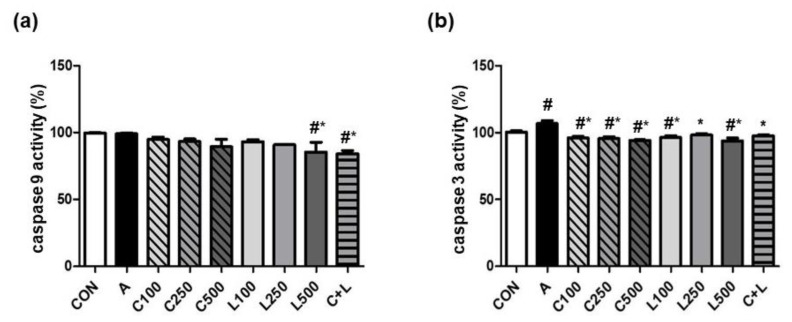
Effects of aspirin, CPC, and/or LBP on apoptotic markers (**a**) caspase 9 activity and (**b**) caspase 9 activity. CON: control group, A: aspirin-induced group, C: CPC treated group, L: LBP treated group. Data are mean ± SD (*n* = 3). # *p* < 0.05 compared with the CON group. * *p* < 0.05 compared with the A group.

Aspirin did not change caspase 9 activity (Figure 4a). A high dose of LBP (500 μg/mL) and C + L treatments significantly decreased caspase 9 activity compared with the control and aspirin-induced groups. Aspirin significantly reduced caspase 3 activity (Figure 4b). The treatment of CPC and/or LBP significantly attenuated caspase 3 activity.

### 3.5. Effects of CPC and LBP on Apoptotic Regulators

**Figure 5:**
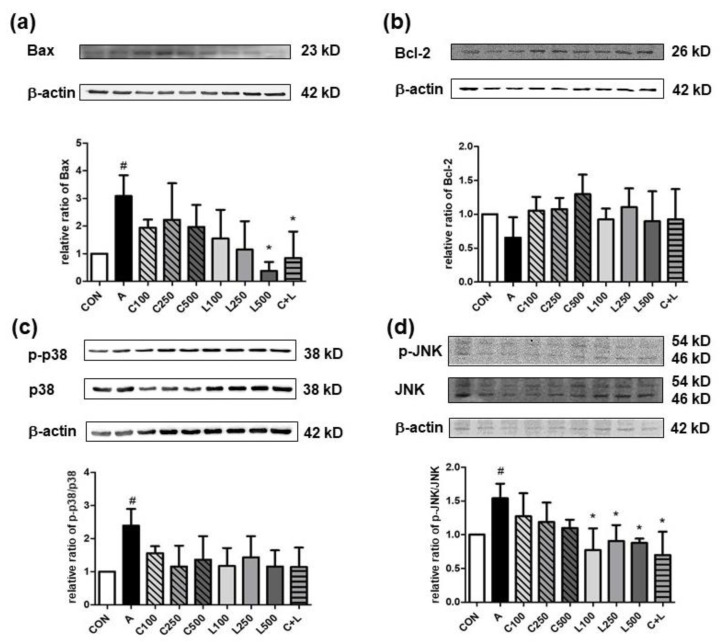
Effects of aspirin, CPC, and/or LBP on the relative ratio of (**a**) Bax, (**b**) Bcl-2, (**c**) phosphorylated p38 (p-p38)/p38, and (**d**) phosphorylated c-Jun N-terminal kinase (p-JNK)/JNK. CON: control group, A: aspirin-induced group, C: CPC treated group, L: LBP treated group. Data are mean ± SD (*n* = 4). # *p* < 0.05 compared with the CON group. * *p* < 0.05 compared with the A group.

Aspirin elevated the pro-apoptotic relative ratio of p-Bax/Bax compared with the control group (Figure 5a). The treated group with LBP at 500 μg/mL or combined CPC and LBP significantly attenuated the relative ratio of p-Bax/Bax. However, no significant differences were shown in the anti-apoptotic relative ratio of p-Bcl-2/Bcl-2 between any two groups (Figure 5b). Aspirin significantly increased the relative ratios of p-p38/p38 (Figure 5c) and phosphorylated c-Jun N-terminal kinase (p-JNK)/JNK (Figure 5d). However, the treatment of CPC or LBP, either alone or a combination of both, could not alter the relative ratio of p-p38/p38 compared with the groups with or without aspirin. The treatment of LBP at all doses and C + L significantly decreased the relative ratio of p-JNK/JNK.

## 4. Discussion

RGM-1 cells were induced lesions by aspirin at a dose of 21 mM for 3 h in this study, and the survival rate was 53–55%. A previous study demonstrated that the cell survival rate of RGM-1 cells treated with 5 mM or 10 mM aspirin for 3 h was less than 80% and 60%, respectively [18]. The survival rate of RGM-1 cells was significantly decreased after cells were damaged by 10 mM aspirin for 18 h [17]. In this study, the survival rate of RGM-1 cells was not altered after cells were incubated with various doses of CPC (30–900 μg/mL) or LBP (50–1000 μg/mL) for 24 h (Figure S1), indicating both CPC and LBP were safe interventions. Additionally, the randomized, double-blind, placebo-controlled human studies found that a high dose of CPC-containing extract (2.3 g/day) for 2 weeks [19] or LBP at a dose of 1.632 g/day for 30 days [20] was well tolerated in adults, and was beneficial for the regulation on certain physical functions.

We demonstrated that aspirin caused significant increases in NF-κB (p65) DNA binding activity ratio and protein expression ratio of p-ERK/ERK. Similarly, indomethacin, an NSAID, could increase p-ERK [21]. Increased p-ERK could activate the IKK-β complex, which in turn activated NF-κB and generated inducible nitric oxide synthase (iNOS), which then led to neutrophil infiltration and inflammation [22]. We speculated that aspirin might activate NF-κB and cause inflammation by activating ERK. Our study showed that the treatment of CPC (100–500 μg/mL) decreased the secretion of TNF-α and IL-6 in RGM-1 cells. Consistent with our finding, CPC at the dose of 50–250 μg/mL led to decreases in elevated TNF-α, IL-6, matrix metalloproteinase-3 (MMP-3), and NO-induced by IL-1β in canine chondrocytes [22]. The intravenous administration of CPC (30 or 50 mg/kg) in rats attenuated carrageenan-induced inflammation by reducing TNF-α and iNOS [23]. A recent in vivo study also showed that spirulina could restore gastric damage caused by aspirin via balancing oxidant and antioxidant system [24]. We showed that the intervention of 100–500 μg/mL LBP for 24 h significantly inhibited the activation of the NF-κB pathway and reduced proinflammatory cytokine IL-6 secretion but not TNF-α secretion in RGM-1 cells. A previous study found that the treatment of LBP at a dose of 600 μg/mL for 24 h followed by lipopolysaccharide-induced damage increased cell survival but suppressed NF-κB protein expression and TNF-α concentration in BV-2 microglial cells [25]. However, LBP could not inhibit TNF-α expression through the NF-κB signaling pathway, which was mediated by toll-like receptors [26]. The secretion of TNF-α in RGM-1 cells in our study was not altered, which could be due to less dosage of LBP compared with that in a previous study mentioned above. An animal study showed that oral feeding of LBP (1 or 10 mg/kg) for 10 h in mice with liver injury induced by carbon tetrachloride for 8 h after 2-h pretreatment of LBP reduced mRNA expression of TNF-α, IL-1β, and iNOS, and decreased NF-κB activity, indicating that LBP may suppress the expression of proinflammatory markers by inhibiting the activation of NF-κB [27].

Aspirin was reported to produce free radicals, which led to the activation of JNK and p38 mitogen-activated protein kinase (MAPK) pathways, altered the balance between pro-apoptotic and anti-apoptotic markers, and further stimulated the release of cytochrome c from mitochondria to activate caspase 9 and caspase 3 [28]. Previous studies showed that anti-apoptotic Bcl-2 protein expression was decreased, and caspase 9 and caspase 3 activities were activated in human gastric epithelial AGS cells damaged by 40 mM aspirin, indicating that aspirin could induce apoptosis in human gastric epithelial cells [29,30]. However, we found that aspirin increased caspase 3 activity, but did not change Bcl-2 protein expression and caspase 9 activity, probably because the dosage of aspirin and/or the incubation time was not enough. The treatment of 10 μM CPC for 4 h attenuated doxorubicin-induced apoptosis in rat cardiomyocytes by suppressing cytochrome c release, Bax protein expression, and caspase 3 activity, and increasing Bcl-2 protein expression and the ratio of Bcl-2 to Bax [31]. The other study demonstrated that CPC decreased apoptosis induced by islet amyloid polypeptide in rat insulinoma β INS-1E cells by reducing oxidative stress and regulating JNK and p38 MAPK pathways [32]. However, our study showed that even caspase 3 activity was inhibited by CPC, protein expression of Bax, Bcl-2, p-p38, and p-JNK was not changed significantly by CPC. The inconsistent results between the previous studies in rat cardiomyocytes or rat insulinoma β cells and the present study in rat normal gastric mucosal cells are probably because of different cell lines and various inducers for cell apoptosis. We found that LBP reduced both caspase 9 and caspase 3 activities by suppressing the activation of JNK. A previous study demonstrated that LBP suppressed apoptosis induced by hydrogen peroxide through increasing Bcl-2 and decreasing Bax protein expression in human lens epithelial SRA01/04 cells [33]. The animal study showed that LBP (40 mg/kg) inhibited apoptosis induced by focal ischemic injury in mice by down-regulating protein expression of cleaved poly(ADP-ribose) polymerase 1, cytochrome c, Bax, caspase 3, and caspase 9, but increasing Bcl-2 protein expression [34]. Additionally, both previous studies mentioned above pointed out that LBP suppressed apoptosis by attenuating oxidative stress [29,30]. Therefore, LBP inhibited apoptosis by modulating pro-apoptotic and anti-apoptotic factors and suppressing oxidative stress.

This study showed that the treatment of LBP at a higher dose of 500 μg/mL might exert the best effects on reducing aspirin-induced inflammation and apoptosis in RGM cells. The anti-inflammatory mechanisms of LBP could be involved in significantly reducing the activation of ERK, thereby attenuating IκBα phosphorylation, NF-κB (p65) DNA binding activity, and finally, proinflammatory IL-6 secretion, and increasing anti-inflammatory IL-10 secretion. The anti-apoptotic action of LBP could be attributed to reducing JNK activation, Bax protein expression, and caspase 3 activity. However, the combined CPC and LBP did not obviously demonstrate additional or synergistic effects on anti-inflammation or anti-apoptosis.

Other natural ingredients or extracts have been observed to exert gastroprotective effects on aspirin-induced damage. An in vitro study found that licoflavone at the dose of 6–12 mg/L had potential gastroprotective effects on antioxidation via enhancing antioxidant enzyme activities and reducing lipid peroxides and on anti-apoptosis via decreasing Bax expression and increasing Bcl-2 expression in human gastric mucosal GES-1 cells damaged by aspirin (18.5 mM) through suppressing ERK1/2 signaling pathway [35], which may have similar properties as CPC and LBP. Araloside A, an active ingredient from *Aralia elata* bark, at the dose of 10–40 mg/kg for 7 days, inhibited gastric damage by aspirin through decreasing gastric activities of H^+^/K^+^-ATPase, apoptotic enzymes, and protein expression of Bax, but enhancing Bcl-2 expression in mice [36]. Artesunate, a derivative from *Artemisia annua L.* as an antimalarial compound, at the dose of 50 or 150 mg/kg has been reported to decrease aspirin-induced gastric lesions in rats by reducing oxidative stress, gastric TNF-α, IL-6, COX-2, and NF-κB expression [37]. Mice given spirulina at the dose of 250–500 mg/kg for 3 days reduced gastric damage induced by aspirin via decreasing lipid peroxidation and inflammatory markers such as TNF-α and COX-2 [38]. Additionally, basil extract at the dose of 100 or 200 mg/kg for 5 days inhibited TNF-α and IL-6 but increased IL-4 and prostaglandin E_2_ in mice serum to protect against gastric injury by aspirin [39]. Bambara groundnut extract is originally from Africa as a common food source and an antioxidant. Pretreatment with Bambara groundnut extract at the dose of 200 or 400 mg/kg for 3 weeks protected against rat gastric ulcer induced by aspirin and pyloric ligation through elevating superoxide dismutase and glutathione peroxidase activities and attenuating malondialdehyde lipid peroxides [40]. Another in vivo study showed that supplementation with 10% pomegranate peel powder for 30 days increased nitric oxide in plasma and decreased gastric TNF-α and COX-2 gene expression in rats with aspirin-induced gastric ulcer [41]. Overall, these results showed that natural ingredients or extracts had gastroprotective actions on aspirin-induced injury by various mechanisms, including antioxidation, anti-inflammation, antacid, anti-apoptosis, and stimulation of gastric mucosal protective factors.

The previous review found that omeprazole combined with rebamipide may be the most effective treatment for aspirin-induced gastric damage compared with several common drugs such as rabeprazole, phosphatidylcholine complex, famotidine, ranitidine bismuth citrate, and lansoprazole from 10 randomized controlled studies [42]. Past studies also compared the gastroprotective effects of natural compounds with medicine [43,44]. Rats were treated with pentagalloyl glucose (50–200 mg/kg) isolated from *Harpullia pendula* pericarp compared with those treated with melatonin, omeprazole, or famotidine (10–40 mg/kg) to explore the gastroprotective properties of the natural compound and medication against gastric injury caused by aspirin [43]. Rats were pretreated with pentagalloyl glucose, melatonin, omeprazole, or famotidine for 1 h, followed by the induction of gastric injury by aspirin for 1 h, and the results showed elevated prostaglandin E_2_ and reduced COX-2 concentrations in the stomach [43]. Another research used ranitidine as a positive control to clarify the effect of *Cyperus rotundus* rhizome extract on gastric lesions caused by aspirin [43]. The results demonstrated that *C. rotundus* rhizome extract at the dose of 250–500 mg/kg suppressed gastric lipid peroxidation and exerted antioxidant action, which showed a similar effect to the anti-ulcer drug ranitidine [44]. However, we did not perform a positive control in this study, and a positive control such as omeprazole can be used to demonstrate whether CPC and/or LBP may have the same effect as the drug in the future study. In addition, the effects of combined natural compounds or extracts with anti-ulcer medicine could be further investigated to clarify whether the combination could reduce the dosage of the anti-ulcer drug in order to not only eliminate the side effects of the drug but also ameliorate aspirin-induced gastric lesions.

## 5. Conclusions

The present study demonstrated that CPC and/or LBP at a high dose of 500 μg/mL reversed gastric injury caused by aspirin via elevating IL-10 and attenuating proinflammatory markers, NF-κB, caspase 3 activity, Bax protein, and the activation of ERK and JNK in RGM-1 cells. Therefore, CPC and/or LBP exert anti-inflammatory effects against damaged gastric RGM-1 cells by aspirin via inhibiting the activation of the ERK signaling pathway. LBP reduces apoptosis by restraining the activation of the JNK signaling pathway.

## Data Availability

Data of the present study are not publicly available. Data are available upon reasonable request and with permission from the authors.

## Supplementary Materials

The following supporting information can be downloaded at: https://www.mdpi.com/article/10.3390/nu14235113/s1, Figure S1: Effects of (a) C-phycocyanin (CPC) or (b) *Lycium barbarum* polysaccharides (LBP) on the cell survival rate of RGM-1 cells.
