# Supplementary material for: C-Phycocyanin and Lycium barbarum Polysaccharides Protect against Aspirin-Induced Inflammation and Apoptosis in Gastric RGM-1 Cells
Source: Nutrients. 2022 Dec 1;14(23):5113. doi: 10.3390/nu14235113 (PMC9736128; doi:10.3390/nu14235113)
Supplement: Supplementary file 1 [file nutrients-14-05113-s001.zip › nutrients-2021095-supplementary.pdf]

*Supplementary Materials*

# C-Phycocyanin and *Lycium barbarum* Polysaccharides Protect against Aspirin-Induced Inflammation and Apoptosis in Gastric RGM-1 Cells

**Yu-Chen Liu [1], Chun-Chao Chang [2,3,4], Hirofumi Matsui [5] and Jane C.-J. Chao [1,4,6,7,*]**

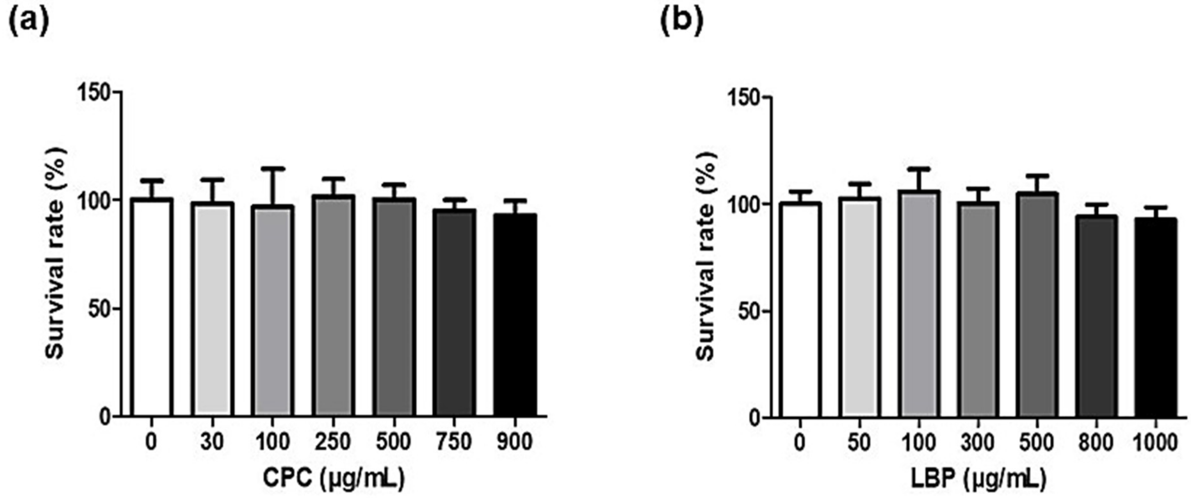


**Figure 1.** Effects of (**a**) C-phycocyanin (CPC) or (**b**) *Lycium barbarum* polysaccharides (LBP) on cell survival rate of RGM-1 cells. CON: control group, A: aspirin-induced group, C: CPC treated group, L: LBP treated group. Data are mean ± SD ($n$ = 6). There were no significant differences between any two groups ($p > 0.05$).
